# Relationships between parent feeding behaviors and parent and child characteristics in Brazilian preschoolers: a cross-sectional study

**DOI:** 10.1186/s12889-018-5593-4

**Published:** 2018-06-07

**Authors:** Sarah Warkentin, Laís Amaral Mais, Maria do Rosário Dias de Oliveira Latorre, Susan Carnell, José Augusto de Aguiar CarrazedoTaddei

**Affiliations:** 1 Department of Pediatrics, Discipline of Nutrology, Federal University of São Paulo (UNIFESP), Rua Loefgreen, 1647, São Paulo, SP CEP: 04040-032 Brazil; 2 School of Public Health, Department of Epidemiology, University of São Paulo (USP), São Paulo, SP Brazil; 3 Department of Psychiatry and Behavioral Sciences, Division of Child & Adolescent Psychiatry, Johns Hopkins University School of Medicine, Baltimore, MD USA

**Keywords:** Children, Feeding practices, Parenting, Diet, Eating behavior

## Abstract

**Background:**

Eating habits formed in early childhood are influenced by parental feeding behaviors, warranting investigation of predictors and correlates of parent feeding. We aimed to describe relationships between parental feeding practices and parent and child characteristics in a sample of Brazilian preschoolers.

**Methods:**

Four hundred and two parents of preschoolers enrolled in private schools of São Paulo and Campinas, Brazil, completed a Brazilian version of the Comprehensive Feeding Practices Questionnaire, as well as questions about parental attitudes, child food intake, other obesity-associated behaviors, and socioeconomic and demographic characteristics. We ran bivariate logistic regression models examining associations between independent variables and each feeding practice. Next, we ran multiple logistic regression models predicting each parental feeding practice.

**Results:**

Greater ‘Restriction for Weight Control’ and ‘Restriction for Health’ were associated with lower maternal education (OR = 2.42 (CI 95% 1.07–5.48) and 2.79 (CI 95% 1.25–6.22), respectively), and with higher concern about child overweight (OR = 2.46, CI 95% 1.64–3.69 for ‘Restriction for Weight Control’, only), while greater ‘Pressure’ was associated with greater concern about child underweight (OR = 2.30, CI 95% 1.53–3.47) and lower maternal BMI (OR = 0.94, CI 95% 0.88–1.00). Greater use of ‘Emotion Regulation/ Food as Reward’ was associated with lower maternal education (OR = 2.22, CI 95% 1.05–4.71). In analyses of positive feeding practices, lesser use of ‘Healthy Eating Guidance’ and ‘Monitoring’ was associated with greater intake of less healthy foods in children (OR = 1.53 (CI 95% 1.01–2.32) and OR = 1.94 (CI 95% 1.27–2.97), respectively), and greater use of screen devices (OR = 1.59 (CI 95% 1.04–2.44) and OR = 1.57 (CI 95% 1.03–2.39), respectively). Lesser use of ‘Healthy Eating Guidance’ was additionally associated with higher maternal BMI (OR = 1.09, CI 95% 1.03–1.16), and lesser use of ‘Monitoring’ with lesser perceived parent responsibility for child feeding (OR = 1.68, CI 95% 1.12–2.52).

**Conclusions:**

Our results demonstrate diverse socioeconomic, anthropometric and behavioral correlates of parent feeding in a large Brazilian sample of parents of preschoolers.

## Background

Eating habits formed in preschool years are likely to persist into later life such that early unhealthy food patterns could have implications for health throughout the life course [1, 2]. These habits are influenced by internal factors, e.g. the child’s own taste and food preferences, and by external factors, such as peers, media, and parents. Parents play a unique role in early childhood because they act as providers, enforcers and role models for children, who are still highly dependent on them [3, 4]. It is particularly important to study parental feeding practices in the preschool years since communication between parent and child becomes more nuanced than at younger ages, while responding to children’s food demands becomes more complex [5].

Parents use a variety of strategies to influence when, what, and how much children eat, and these may have maximal impact when children are young, and in constant contact with their parents [6]. Parental feeding practices are an attempt to maintain or modify children’s eating habits and weight status. Some of these efforts achieve the parent’s intended goals. For example, the use of feeding practices such as making healthy foods available, and modelling the consumption of healthy food, have been shown to facilitate healthy eating behaviors in young children [7]. Additionally, a recent prospective observational study suggested that monitoring a child’s intake of high energy-dense foods at age 2 was associated with a healthier weight status one year later [8]. Kröller & Warschburger (2008) found that mothers with higher education reported using more monitoring regarding children’s food intake [9].

On the other hand, certain behaviors may have unintended consequences. Coercive feeding practices, such as ‘Restriction’, may promote overeating in children, increasing the risk for subsequent problems with eating and energy balance [10]. Restricting children’s food intake is correlated with parental knowledge and overvaluation of the child’s weight and body shape [11], and has been associated with several demographic characteristics of parents, including lower maternal age and education [12]. Pressuring the child to eat, usually during mealtimes, is also a common practice, which may override children’s internal cues of hunger and satiety [1], and associate with eating beyond satiety, since internal satiety cues are ignored [1, 13], increased intake of unhealthy foods in early childhood [9, 14], and a negative atmosphere at mealtime [4].

Given the potential relevance of parental feeding practices in young children for future nutrition disorders, it is necessary to investigate potential predictors of these practices across different contexts and environments. Child overweight is increasing in developing countries such as Brazil, with national surveys showing an increase of 160% from 1989 to 2006 (9.4% per year) [15], accompanied by large increases in availability and variety of food products promoting ultra-processed food consumption [16]. Since parents are the first nutritional educators for their children, the investigation of parental feeding practices is essential, yet currently understudied, in Brazil.

The present study therefore aimed to test associations between multiple parental feeding practices and diverse parent and child characteristics in a large sample of preschoolers enrolled in Brazilian private schools. To ensure coverage of a wide range of influential feeding strategies we used a validated Brazilian version [17] of the Comprehensive Feeding Practices Questionnaire (CFPQ) [18]. As parental feeding practices have been associated with a number of parent characteristics such as maternal age [6, 12], education [12], weight status [6, 12], socioeconomic status [6], and child characteristics such as gender [14], child age [12, 14], weight status [1], daily screen time [14] and food intake [14, 17], we included each of these factors as independent variables.

In accordance with the literature, our primary hypotheses were that the use of potentially negative and non-nutritive feeding practices (‘Restriction for weight control’, ‘Restriction for health’, ‘Pressure’, ‘Emotion regulation/ food as reward’) would be associated with lower parental educational levels, higher parental weight status, less healthy eating habits in children (e.g. greater intake of ultra-processed foods), and higher child weight status. We additionally hypothesized that for positive feeding practices (‘Healthy Eating Guidance’ and ‘Monitoring’), lower use would be associated with lesser parental education, less healthy eating habits (e.g. lesser intake of healthier foods such as fruits and vegetables), and higher child weight status. We expected parental restriction to be predicted by parents’ concerns about child overweight, and parental pressure to eat to be predicted by concerns about child underweight.

## Methods

### Study design

This research was conducted as part of a broader cross-sectional study, the ‘*Estudo de Práticas Alimentares*’ (EPA), which aimed to adapt and validate the CFPQ for a Brazilian sample [17], as well as to investigate relationships with parent and child characteristics.

### Participants

Participants were middle- and high-income parents of 2-to-5-year-olds recruited from April through June 2014 from private schools in the cities of São Paulo and Campinas. Children with diseases that were related to nutrition and/or could influence parental feeding practices (e.g. cystic fibrosis, diabetes or autism), children who had a younger sibling who was participating (in order to avoid sample unit duplication), and not being within the eligible age group were excluded.

To estimate the sample size, we based power calculations on an effect of maternal weight status (overweight i.e. ≥25.0 Kg/m^2^ versus non-overweight i.e. < 25.0 Kg/m^2^), assuming a proportion of 65 and 45% for the use of more coercive parental feeding practices among overweight and non-overweight mothers, respectively. After an addition of 10% for anticipated losses, this analysis yielded a minimum required sample size of 310 respondents to achieve a probability of 0.05 for type I error and 0.20 for type II error.

### Procedures

The first phase of the EPA involved validation of the CFPQ in Brazilian preschool [17] and school-aged children [19]. This resulted in a slightly modified, reduced instrument, with a new factor structure (6 factors and 43 items), that was used for the current study. The validated instrument showed good validity and reliability (‘Restriction for Weight Control’: α = 0.84; ‘Restriction for Health’: α = 0.88; ‘Emotion Regulation/Food as Reward: α = 0.74; ‘Pressure’: α = 0.72; ‘Healthy Eating Guidance’: α = 0.83; ‘Monitoring’: α = 0.86) [17]. Before participating in the survey, caregivers each gave written consent. Survey packets containing questionnaires and instructions requesting completion within two weeks by a parent were left in each classroom. In one of the schools, the questionnaires were administered and completed by parents before a parents and teachers meeting. More details on the data collection procedure are described elsewhere [17]. This research received ethical approval from the Federal University of São Paulo (UNIFESP) ethics committee.

### Measures

The Brazilian version of the CFPQ includes the following factors: ‘Restriction for Weight Control’ (RWC, the degree to which a parent restricts their child’s food intake in order to limit weight gain – 7 items), ‘Restriction for Health’ (RH, the degree to which a parent restricts their child’s food intake with the motivation of promoting healthy eating rather than influencing body weight – 5 items), ‘Emotion Regulation/Food as Reward’ (ER/FR, the degree to which a parent uses food as reward for desired behavior in their child, or to regulate emotion – 6 items), ‘Pressure’ (P, the degree to which a parent pressures the child to eat – 4 items), ‘Healthy Eating Guidance’ (HEG, parents’ facilitation of a healthy eating environment, including teaching, modeling and child’s involvement in food intake – 16 items), and ‘Monitoring’ (M, the degree to which the parent keeps track of their child’s consumption of unhealthy foods – 4 items). Responses are given on a 5-point Likert scale ranging from ‘never’ to ‘always’ for frequency items, and from ‘totally disagree’ to ‘totally agree’ for statements [17]. For each CFPQ sub-scale, we summed the response values of each item.

Information about socioeconomic and demographic characteristics, as well as parent and child anthropometric measures, were reported by the parents (376 (93.54%) mothers and 26 (6.46%) fathers). Parental attitude scales were derived from the Child Feeding Questionnaire (CFQ) [20]. ‘Concern about Child Overweight’ (3 items) and ‘Perceived Responsibility for Child Feeding’ (3 items) scales were taken directly from the CFQ. ‘Concern about Child Underweight’ (3 items) was adapted from the CFQ by changing the words ‘overweight’ to ‘underweight’, and ‘diet’ to ‘eat more’ [18]. Responses were given on a 5-point Likert scale ranging from ‘never’ to ‘always’, and scale scores were generated by calculating the mean of each factor (Table 1).

**Table 1:** Demographic and anthropometric characteristics of children (*n* = 402)

	M (SD)^a^	n (%)
Parent characteristics		
Respondent		
Mother		376 (93.54)
Father		26 (6.46)
Parent age	36.42 (4.69)	
Mother education		
< college complete		**31 (7.71)**
college complete		371 (92.29)
Father education		
< college complete		**49 (12.34)**
college complete		348 (87.66)
Mother BMI^b^	23.22 (3.44)	
Mother weight status^c^		
Overweight/obese		**95 (23.99)**
Normal weight		286 (72.22)
Underweight		15 (3.79)
Father weight status^c^		
Overweight/obese		**277 (71.76)**
Normal weight		108 (27.98)
Underweight		1 (0.26)
Perceived responsibility for child feeding		
Never/seldom/half of the time/most of the time		**180 (44.89)**
Always		221 (55.11)
Concern about child overweight		
Concerned/fairly concerned/very concerned		**205 (51.38)**
Unconcerned/a little concerned		194 (48.62)
Concern about child underweight		
Fairly concerned/very concerned		**205 (51.12)**
Concerned/a little concerned/unconcerned		196 (48.88)
Child characteristics		
Sex		
Male		**195 (48.51)**
Female		207 (51.49)
BMI z-score^d^		
Overweight/obese/extremely obese		**97 (24.81)**
Normal weight/ underweight/ extremely underweight		294 (74.19)
Ultra-processed food intake^e^		
Frequent consumption		**259 (64.43)**
Infrequent consumption		143 (35.57)
Traditional food intake^f^		
Infrequent consumption		**244 (60.70)**
Frequent consumption		158 (39.30)
Screen time (per day)		
> 2 h		**152 (37.81)**
≤2 h		250 (62.19)

Bold indicates the risk category

^a^ mean (standard deviation); ^b^ BMI: body mass index.; ^c^ BMI cut-off ≥25.0 Kg/m^2^ for overweight/obese; ^d^ BMI z-score cut-off ≥ + 1 z-score for overweight/obese/extremely obese; ^e^ Frequent consumption = n (%) with score ≥ 33rd centile for variable (score ≥ 1.69) i.e. on average, consumed one or more of these foods within the last 7 days; ^f^ Infrequent consumption: n(%) with score ≤ 66th centile for variable (score ≤ 4.83) i.e. on average, did not consume any of these foods within the last 7 days

Dietary data regarding child food intake for the last seven days was gathered using a Food Frequency Questionnaire (FFQ) specially designed for this population, based on the foods most frequently consumed in the Brazilian population, and incorporating ultra-processed foods known to be associated with obesity [21]. Responses were given on a 5 point scale with the following options: 1 = did not consume, 2 = consumed 1–2 times/week, 3 = consumed 3–4 times/week, 4 = consumed 5–6 times/week, 5 = consumed every day. Using this data we created variables representing frequencies of intake for traditional foods (i.e. beans, meat and eggs, fruits, milk and dairy, vegetables and grains) and ultra-processed foods (i.e. artificial juice, breakfast cereal, chips, chocolate milk, crackers/biscuits/cakes with and without stuffing, dairy desserts, fast food, ice-cream/popsicle, instant noodles, processed meat, soft drink and sugary snacks) [22]. For each food category, scores for all food items were summed, divided by the total number of items, and then transformed to a 1–100 scale. Additionally, we asked parents to provide information about child’s daily screen time using the following response options: 1 = no use, 2 = less than 2 h, 3 = 3–4 h, 4 = 5–6 h, 5 = more than 6 h.

### Data treatment and statistical analysis

In order to a) choose an approach that was robust to the non-normal distributions of parent feeding behavior scores (confirmed using Kolmogorov-Smirnov testing), b) facilitate interpretation of the studied associations by quantifying the risk of defined levels of each parent and child characteristic being associated with defined quantities of parental feeding behavior, and c) promote comparison between factors by presenting associations using a shared scale for risk magnitude, we chose to dichotomize our multiple independent and dependent variables and apply logistic regression.

For CFPQ sub-scales (dependent variables), we implemented a median split, and considered higher (above median) scores on negative/non-nutritive feeding practices (i.e. RWC: > 25.00 (of range 0–96.43; RH: > 70.00 (of range 0–100); P: > 62.50 (of range 0–100); ER/FR: > 12.50 (of range 0–75), and lower (below median) scores on positive feeding practices (i.e. HEG: < 87.50 (of range 46.87–100) and M: < 98.75 (of range 0–100)), as indicating higher obesity risk.

For maternal weight status we split the sample into those who were Overweight/Obese (i.e. BMI ≥ 25) and those who were normal-weight. Child weight status was determined based on World Health Organization (WHO) guidelines, such that those children with z-score < − 3 were designated ‘Extremely underweight’, those with z-score ≥ − 3 but < − 2 ‘Underweight’, those with z-score ≥ − 2 but < + 1 ‘Normal weight’, those with z-score ≥ + 1 but < + 2 ‘Overweight’, those with z-score ≥ + 2 but < + 3 ‘Obese’ and those with z-score ≥ + 3 ‘Extremely obese’ [23]. We then split the sample into those who were Overweight/ Obese/ Extremely obese) vs. those who were Normal weight/ Underweight/ Extremely underweight.

For parental attitude scales, we based dichotomization on the median for each scale, i.e., a score < 15.0 for Perceived Responsibility for Child Feeding, which corresponded to a mean response of Never, Seldom, Half of the time, or Most of the time across the scale items a score, a score ≥ 7.0 for Concern about Child Overweight, which corresponded to a mean response of ‘Concerned’, ‘Fairly concerned’ or ‘Very concerned’ across the scale items, and a score ≥ 9.0 for Concern about Child Underweight, which corresponded to a mean response of ‘Fairly concerned’ or ‘Very concerned’ across the scale items.

For the FFQ data (independent variables), we implemented cut-off values based on dual consideration of the data distribution and the associated response values. For ultra-processed foods, we used a cut-off value of 33rd centile (i.e score ≥ 1.69), considering values equal or above it as indicating ‘frequent consumption’, (approximately corresponding to consumption of specified items from the specified ultra-processed food categories, on average, 1–2 times a week or more), and values below indicating ‘infrequent consumption’ (approximately corresponding to no consumption of any ultra-processed item within the last 7 days) (Table 1). For traditional foods we chose a cut-off of 66th centile with scores equal to or under 66th centile (i.e. score ≤ 4.83) considered as indicating, on average, ‘infrequent consumption’ (approximately corresponding to consumption of specified items from the specified food categories, on average, 5–6 times a week or less), and scores above 66th centile considered as indicating ‘frequent consumption’ (approximately corresponding to consumption of each item 7 days a week). This centile-based approach insured that we had sufficient numbers in each group for our logistic regression models to be valid.

We then ran a series of bivariate logistic regression analyses examining associations between each independent variable and each parental feeding practice. Next, to establish which variables were associated with parental feeding practices, independently of other confounders, we ran multiple logistic regression models for each parental feeding practice using the Stepwise Forward entering method. A criterion of *p* ≤ 0.20 was used to identify independent variables for initial entry into these models, then, after using the Stepwise Forward enter method, only variables with *p*-value less than 0.05 were considered significant and retained for the final model [24]. All data were analyzed using Stata version 14.0 [25].

## Results

From the total of 46 contacted schools, 14 agreed to participate in the study. We distributed 996 questionnaires and 448 (44.98%) of them were retained for analysis. Of the 548 other questionnaires, 526 were not returned, 18 had missing data on the CFPQ, and 4 had numerous incomplete answers including essential items concerning family characteristics. The final sample size was 402. A more detailed description of the excluded participants is given elsewhere [17].

Descriptive analysis (Table 1) revealed that the majority of parents were highly educated, with only 10% of the respondents classifying their education as less than college. The vast majority of questionnaires were completed by the mother of the child (93.54%) and, according to calculated BMI, mothers were mostly normal weight (76%). Parents reported greater concern about their child becoming overweight (51.62%), than about their child becoming underweight (38.65%).

Table 2 shows the results of bivariate logistic regression analyses for all independent and dependent variables. All variables with *p* ≤ 0.20 were deemed eligible to enter the final model, shown in Table 3. In addition to the dichotomized variables, in order to confirm that our results were not unduly influenced by our analysis choice, we also re-conducted analyses using continuous independent variables where possible (i.e. for child and mother weight, and child and mother age). Results were unchanged with the exception of associations that emerged between ‘Pressure’ and maternal BMI (OR = 0.94 (CI 95% 0.88–0.96, *p* = 0.034), and ‘Healthy Eating Guidance’ and maternal BMI (OR = 1.10 (CI 95% 1.03–1.17, *p* = 0.002)). We therefore included this additional analysis in Table 2 and considered maternal BMI for the multiple logistic regression models.

**Table 2:** Results of bivariate logistic regression models showing relationships between each parent and child characteristics, and each parent feeding practice (*n* = 402)

Variables	Risk Category	Negative/ Non-nutritive feeding practices				Positive feeding practices	
		Restriction for Weight Control (RWC)	Restriction for Health (RH)	Pressure (P)	Emotion Regulation/Food as Reward (ER/FR)	Healthy Eating Guidance (HEG)	Monitoring (M)
OR (CI 95%)							
Parent characteristics							
Parent age^a^	–	1.01 (0.97–1.06)	1.03 (0.99–1.07)	1.02 (0.98–1.07)	0.97 (0.93–1.01)	1.00 (0.96–1.04)	0.98 (0.95–1.03)
Mother education	<college complete	**2.68 (1.22–5.87)**	**2.83 (1.29–6.19)**	0.98 (0.47–2.04)	**2.21 (1.05–4.61)**	0.70 (0.34–1.44)	2.13 (0.98–4.65)
Mother BMI^b^	–	0.98 (0.92–1.03)	1.02 (0.96–1.08)	**0.94 (0.88–0.96)**	0.99 (0.94–1.05)	**1.10 (1.03–1.17)**	1.04 (0.98–1.10)
Mother weight status	Overweight/obese	0.96 (0.60–1.52)	1.58 (0.99–2.51)	0.65 (0.41–1.05)	0.84 (0.53–1.34)	**1.61 (1.00–2.57)**	1.22 (0.77–1.93)
Perceived responsibility for child feeding	Never/seldom/half of the time/most of the time	1.31 (0.88–1.94)	**0.66 (0.45–0.99)**	0.95 (0.64–1.41)	1.17 (0.79–1.74)	1.25 (0.84–1.86)	**1.77 (1.19–2.63)**
Concern about child overweight	Concerned/ fairly concerned/ very concerned	**2.54 (1.70–3.80)**	**1.52 (1.02–2.25)**	1.12 (0.75–1.66)	1.18 (0.80–1.76)	1.06 (0.72–1.58)	1.00 (0.68–1.49)
Concern about child underweight	Fairly concerned/ very concerned	1.14 (0.77–1.68)	1.37 (0.92–2.03)	**2.30 (1.54–3.46)**	1.11 (0.75–1.65)	0.98 (0.67–1.46)	0.79 (0.54–1.17)
Child characteristics							
Sex	Male	0.87 (0.59–1.28)	1.37 (0.93–2.03)	1.15 (0.78–1.72)	0.94 (0.64–1.40)	1.26 (0.85–1.86)	1.25 (0.84–1.85)
Weight status	Overweight/obese/extremely obese	**1.71 (1.09–2.67)**	1.43 (0.92–2.24)	**1.63 (1.03–2.59)** ^**c**^	1.07 (0.68–1.67)	1.28 (0.82–2.01)	1.28 (0.82–2.00)
Ultra-processed food intake	Frequent consumption	0.95 (0.63–1.42)	1.36 (0.90–2.04)	1.02 (0.68–1.54)	1.36 (0.90–2.06)	1.39 (0.92–2.09)	**2.10 (1.39–3.19)**
Traditional food intake	Infrequent consumption	1.24 (0.83–1.86)	1.47 (0.99–2.20)	0.99 (0.66–1.49)	1.50 (0.99–2.25)	**1.69 (1.13–2.53)**	1.37 (0.92–2.05)
Screen time (per day)	> 2 h	1.17 (0.78–1.75)	1.33 (0.86–1.98)	1.03 (0.69–1.55)	1.42 (0.95–2.13)	**1.81 (1.20–2.72)**	**1.70 (1.13–2.56)**

*OR* odds ratio, *CI* confidence interval, *p p*-value. Results in bold are significant at *p* ≤ 0.05. For this analysis, we defined risk categories as follows. HEG: < 87.50 (possible range 46.87–100); M: < 98.75 (possible range 0–100); RWC: > 25.00 (possible range 0–96.43); RH: > 70.00 (possible range 0–100); *P*: > 62.50 (possible range 0–100); ER/FR: > 12.50 (possible range 0–75).

^a^ Age of respondent parent as continuous variable

^b^ Mother BMI as continuous variable

^c^ Risk category in BMI z-score: “normal weight”

**Table 3:** Results of multiple logistic regression models showing significant associations between parent and child characteristics for each parent feeding practice

Variables	Risk Category	Negative/Non-nutritive feeding practices				Positive feeding practices	
		Restriction for Weight Control (RWC) (*n* = 399)	Restriction for Health (RH) (*n* = 402)	Pressure (P) (*n* = 395)	Emotion Regulation/Food as Reward (ER/FR) (*n* = 402)	Healthy Eating Guidance (HEG) (*n* = 396)	Monitoring (M) (*n* = 401)
OR (CI 95%)							
Parent characteristics							
Mother education	<college complete	2.42 (1.07–5.48)	2.79 (1.25–6.22)	–	2.22 (1.05–4.71)	–	–
Mother BMI^a^	–	–	–	0.94 (0.88–1.00)		1.09 (1.03–1.16)	–
Perceived responsibility for child feeding	Never/seldom/ half of the time	–	–	–	–	–	1.68 (1.12–2.52)
Concern about child overweight	Concerned/ fairly concerned/ very concerned	2.46 (1.64–3.69)	–	–	–	–	–
Concern about child underweight	Fairly concerned/ very concerned	–	–	2.30 (1.53–3.47)	–	–	–
Child characteristics							
Ultra-processed food intake	Frequent consumption	–	–	–	–	–	1.94 (1.27–2.97)
Traditional food intake	Infrequent consumption	–	–	–	–	1.53 (1.01–2.32)	–
Screen time (per day)	> 2 h	–	–	–	–	1.59 (1.04–2.44)	1.57 (1.03–2.39)

*OR* odds ratio, *CI* confidence interval, *p* p-value. All models adjusted for child sex. Risk categories defined as follows. HEG: < 87.50; M: < 98.75; RWC: > 25.00; RH: > 70.00; *P*: > 62.50; ER/FR: > 12.50

^a^Mother BMI as continuous variable

Final multiple logistic regression models for each CFPQ factor, adjusted for child sex, are presented in Table 3. In analyses of negative/non-nutritive feeding practices, both ‘Restriction for Weight Control’ and ‘Restriction for Health’ were associated with lower maternal education (RWC: OR = 2.42 (CI 95% 1.07–5.48, *p* = 0.034), RH: OR = 2.79 (CI 95% 1.25–6.22, *p* = 0.013). ‘Restriction for Weight Control’ was additionally associated with greater concern about child overweight (OR = 2.46 (CI 95% 1.64–3.69, *p* < 0.001)). Parents that used more ‘Pressure’ were 2.3 times more likely to be concerned about child underweight and lower mother BMI (OR = 0.94 (CI 95% 0.88–1.00, *p* = 0.038)). Greater use of the feeding practice ‘Emotion Regulation/ Food as Reward’ was associated with lower maternal education (OR = 2.22 (CI 95% 1.05–4.71, *p* = 0.038)).

For positive feeding practices, lesser use of ‘Healthy Eating Guidance’ was associated with infrequent consumption of traditional foods (OR = 1.53 (CI 95% 1.01–2.32, *p* = 0.046)), while, lesser use of ‘Monitoring’ was associated with greater consumption of ultra-processed foods (OR = 1.94 (CI 95% 1.27–2.97, *p* = 0.002)). The lesser use of both of these feeding practices were associated with screen time > two hours per day (HEG: OR = 1.59 (CI 95% 1.04–2.44, *p* = 0.034), M: OR = 1.57 (CI 95% 1.03–2.39, *p* = 0.036)). Finally, lower scores on ‘Healthy Eating Guidance’ were associated with greater maternal BMI (OR = 1.09 (CI 95% 1.03–1.16, *p* = 0.004)) and lower use of ‘Monitoring’ was associated with lower perceived responsibility for child feeding (OR = 1.68 (CI 95% 1.12–2.52, *p* = 0.012)).

## Discussion

This study of parental feeding practices in a large sample of Brazilian preschoolers revealed that a number of both positive and negative/non-nutritive feeding behaviors were significantly associated with selected parent and child characteristics, partially confirming our specified hypotheses. Below we discuss results specifically relating to our hypotheses.

For our first hypothesis, that the use of negative/non-nutritive feeding practices would be associated with lower parental educational levels, less healthy eating habits, and higher child weight status, results were partly consistent. Specifically, these practices were indeed used more often by parents with lower education levels. In fact, less educated mothers were almost 2.42 times more likely to employ ‘Restriction for Weight Control’ and almost 2.8 times likely to use ‘Restriction for Health’. This association of greater restriction with lower parental education is consistent with a longitudinal study in the US [26] and a cross-sectional study in the UK [27], and suggests that educational interventions promoting effective feeding practices may benefit from targeting mothers with lower education levels. Less educated parents also had a greater tendency to use food to regulate emotions or as reward, as has been found in other studies (use of food as reward: [28, 29]; emotional feeding [30]). Since instrumental feeding has been associated with negative nutritional outcomes in children [10, 31], attempts to curtail the use of these strategies among less educated mothers may be beneficial. The association with only mother education, not father education, may reflect that, in Brazil, the mother is still the main parent responsible for feeding and educating children at home [32]. Wealthier mothers in Brazil with a higher educational level and monthly income have more access to information and care of their children, as demonstrated in a nationally representative study [33]. Contrary to our first hypothesis, we did not find any associations between negative/non-nutritive feeding practices and less healthy eating habits in children. Notably, greater use of ‘Restriction for Weight Control’ was associated with greater child weight using bivariate logistic regression, but this effect did not remain in multiple logistic regression models including other influential variables.

Our second hypothesis, that for healthier feeding practices (‘Monitoring’, ‘Healthy Eating Guidance’), lower use would be associated with lower maternal education, less healthy eating habits and higher child weight status, was partially confirmed. Lower use of ‘Monitoring’ did not show an association with lower maternal education. However, the use of practices such as serving as a model for healthy eating and teaching about the nutritional value of foods (captured in the ‘Healthy Eating Guidance’ factor) has previously been associated with parental knowledge about these topics [34] and could be emphasized in nutritional education programs. In addition, parents with lower scores on both ‘Monitoring’ and ‘Healthy Eating Guidance’ reported that their children consumed fewer healthy (traditional) foods. This was also demonstrated in a recent study, which found that lower use of ‘Healthy Eating Guidance’ was related to lesser intake of fruits and vegetables, as well as greater intake of unhealthy snacks and beverages [35]. It is well-established that healthy eating promoting parental practices such as modelling and providing fruits and vegetables at home – both of which were encompassed within our ‘Healthy Eating Guidance’ factor – increases preschoolers’ consumption of healthier foods [4, 6, 7, 36]. Further, in our own data, lower use of ‘Monitoring’ was associated with greater consumption of ultra-processed foods. Consistent with this finding, ‘Monitoring’ has repeatedly been positively associated with healthier outcomes, such as lower consumption of sweet drinks and non-core foods [35], and negatively associated with unhealthy eating [37]. It is possible that parents who reported higher ‘Monitoring’ levels also perceived themselves as more responsible for child feeding, suggesting a greater engagement with child nutrition [28]. Contrary to our hypotheses, we did not find any association between positive feeding practices and lower child weight. This may have been because the preschoolers in our sample had not been exposed to these beneficial strategies long enough to have an impact on their weight status. Longitudinal study designs are necessary to test for such long-term impacts. Notably, studies examining positive, rather than negative, feeding practices are scarce and our results therefore contribute to the wider literature on parent feeding by suggesting these practices may help to decrease children’s ultra-processed food intake [22]. In Brazil, to our knowledge, there are no reports of potential consequences of a variety of positive feeding behaviors among preschoolers; the present study therefore contributes unique information that could be particularly relevant for development of health interventions in Brazil.

We also found support for our third hypothesis, that parental feeding strategies would be predicted by parents’ concerns about child weight. Specifically, we found that greater concern about child overweight increased the likelihood of using ‘Restriction for Weight Control’. This is consistent with other work suggesting that parents who restrict their child’s food intake may do so in response to parental concerns about child overweight [1, 5, 38–40], and confirms that this relationship is also present in the Brazilian population. This is likely due to increased recognition of the dangers of overweight, and the presence of stigma associated with excessive weight, across most modern societies. According to Damiano et al. (2016), maternal lack of knowledge about parenting strategies and overevaluation of child’s body shape and weight would also be predictors in the use of restrictive feeding practices [11]. A previous study using the CFPQ also found that parental concern about child overweight was related to higher restriction of child’s eating for both weight and health reasons [41]. In addition, we found that greater parental concern about child underweight was positively associated with pressuring. Consistent with this finding, pressuring has been previously associated with both lower child weight [6, 40], and higher concern about child undereating and underweight [40]. Parental concern about their child undereating has also been associated with identification of their child as being a picky or fussy eater [1, 5]. Notably, our scale assessing concern about underweight also included one item assessing concern about undereating. Our findings are therefore consistent with a parental response interpretation whereby concern is a response to a child who eats less than the parent deems suitable, and is picky about which items he or she eats. Importantly, parental perception of a small appetite may not necessarily indicate that the child actually eats too little for optimal health; it could instead reflect difficulty on the part of parents in assessing appropriate portion sizes for preschoolers, or a lack of security regarding their child’s ability to self-regulate their food intake [5].

Finally, regarding the last hypothesis, we did not find any association between greater risk of using negative/non-nutritive feeding practices among overweight/obese mothers – instead, we found that greater pressure was associated with lower maternal weight. The former finding could be driven by mothers with lower weight being more conscious of low intake and weight in children, and, conversely, by mothers with higher weight being more aware of increased risk of overweight in their children and therefore abstaining from this practice [42]. We also found that lower ‘Healthy eating guidance’ was associated with greater maternal weight. This corroborates the results of another study finding that the lack of positive practices, of ‘Modelling’ and ‘Teaching about nutrition’ were associated with greater maternal weight [28], and suggests a potential route for familial transmission of obesity risk.

Our use of a validated measure to assess parental feeding practices in a relatively large sample of preschoolers is a strength of this study, as is our use of a Brazilian population. Only a small number of studies have thus far investigated parental feeding practices in Brazil and, since parents’ feeding practices may be influenced by cultural norms and other sociocultural factors, investigating them across different populations is essential [28, 37]. Our study also had some limitations. First, the data is cross-sectional and thus causal relations cannot be inferred, so interpretation of the results should be made with caution. Second, the sample was relatively well educated. The fact that we observed differences in negative feeding practices by education suggests that, despite this limitation, we did have sufficient variance to detect some important education-related effects. However the lack of variability in education could also have limited our ability to see significant education-related differences in positive feeding practices. Our findings may not replicate in lesser educated Brazilian families. Third, our use of sample-based median splits to dichotomize the parent feeding variables, although statistically prudent due to non-normality, does not allow us to infer practical or theoretical significance of scoring ‘high’ or ‘low’. To rigorously establish clinically-meaningful cut-offs would require reference to a much larger evidence base including longitudinal and experimental studies that could conclusively attribute negative child outcomes to certain levels of the named parent feeding strategies. The literature does not currently allow such an endeavor. Fourth, participants could have answered in a socially desirable way to questions regarding their child food intake and their behaviors – a disadvantage common to all parent-report survey research. The FFQ we used here was not validated previously. Dietary assessment in young children is challenging in general, and relies on dietary information provided by the parents, which is prone to error. Notwithstanding, FFQs have been shown to be effective at ranking children according to their dietary patterns [43]. In addition, all the obtained information was self-reported by parents, which could have led to recall errors. Anthropometric data for parents and children was reported rather than measured; this may have somewhat limited our ability to detect reliable effects of parent and child weight. However, the majority (about 70%) of the anthropometric information provided by parents was derived from pediatrician/medical reports or measured at home (data not shown), and parents of children of this age group are likely to be more aware of their child’s growth data due to contact with health professionals for checks on development. Finally, since our main focus was to analyze variables associated with and potentially predictive of parental feeding practices (which are subjective variables), self-reported weight and height information were arguably more directly relevant to the use of these practices at home.

## Conclusions

To summarize, our results suggest that lower use of positive feeding practices is associated with child characteristics such as unhealthy food intake and excessive screen time, and parental lack of responsibility about child feeding. In contrast, characteristics such as parental education may influence the use of negative/non-nutritive feeding practices, which could lead to unhealthy health or weight outcomes in children. Parental concern about child weight status (under- and overweight) also predicted negative/non-nutritive feeding practices. Since preschool children are undergoing rapid evolution in their eating habits and emotional relationships with food, which may go on to predict their eating and weight gain in later childhood and adulthood, interventions informed by the risk factors we identified here may be beneficial for the Brazilian population. For example, an educational intervention highlighting potential consequences of negative/non-nutritive feeding practices and describing how to implement positive feeding practices, could be targeted at lesser educated or obese mothers. Such an intervention could help to promote healthy eating habits and prevent future nutritional disorders.

## Abbreviations

BMI: Body Mass Index
CFPQ: Comprehensive feeding practices questionnaire
CFQ: Child feeding questionnaire
ER/FR: Emotion regulation/food as reward
FFQ: Food frequency questionnaire
HEG: Healthy eating guidance
M: Monitoring
P: Pressure
RH: Restriction for Health
RWC: Restriction for weight control
